# Special Issue “Biomembranes and Biomimetic Membranes—From Model Analysis to ‘In Vivo’ Study”

**DOI:** 10.3390/membranes12020221

**Published:** 2022-02-14

**Authors:** Wojciech Smułek, Monika Rojewska

**Affiliations:** Institute of Chemical Technology and Engineering, Poznan University of Technology, Berdychowo 4, 60-965 Poznań, Poland; monika.rojewska@put.poznan.pl

Membrane processes are one of the key factors influencing the function of living cells. Understanding how biomembranes function, and being able to regulate their functions, is crucial from the perspective of advancing medicine and biology. Moreover, the conclusions drawn from such research are directly applicable in various biotechnological processes, where membranes that mimic the properties of real biological membranes can be used.

Since biological membranes are complex systems, it is a great challenge to represent them in laboratory conditions. Biomimetic systems, which are of increasing interest to scientists, not only require sophisticated methods of preparation but also appropriately specialized tools for observation and investigation. It also requires cooperation of specialists from various fields, especially biologists, microbiologists, chemists, and physicists.

The aim of our Special Issue “Biomembranes and Biomimetic Membranes—from Model Analysis to ‘In Vivo’ Study” was precisely to provide an opportunity for authors from different disciplines to present their research on processes occurring in biomembranes and the biomimetic membranes that reproduce them. We are very pleased that the published papers show a broad spectrum of approaches to the topic.

Plant cell membranes have been studied by Tang et al. [1]. They wanted to improve their understanding of the nature of phytosterol–phospholipid interaction and find out how the phytosterols modulate the mechanical properties of lipid membranes. The linking of optical microscopy and computational microscopy (MD simulation) is particularly effective when studying the mechanisms of lipid conformation dynamics, nanodomain formation, phase separation, and membrane homeostasis. Another interesting study is that of Najib et al. [2] who focused on issues concerning lipid vehicles and their interactions with membranes. They investigated the uptake of different oily vehicles and blends of oils into silicone membrane and its effect on the diffusion of model permeants. They concluded that oils have significant potential to modify the barrier properties of membranes.

The Special Issue closes with a review paper by Rojewska et al. [3] on the use of monolayer experiments undertaken to investigate the impact of selected antibiotics on components of biomembranes, with particular emphasis on the role and content of individual phospholipids and lipopolysaccharides (LPS). The authors noticed that, in combination with spectroscopic techniques, the Langmuir method may help to establish preferred antibiotic orientations and the depths of its insertion into the cell membrane. It brings valuable information because the transportation of antibiotics through membranes is frequently a crucial factor regulating their biocidal effectiveness. 

At the end, we would like to express our gratitude to all the authors of the published papers. Without their commitment, experience, and work, our Special Issue would not have come into existence.

## Data Availability

Not applicable.
